# Long term study on blood glucose levels in wintering great tits *Parus major* in sites differing in artificial food availability

**DOI:** 10.1038/s41598-025-86190-w

**Published:** 2025-01-20

**Authors:** Adam Kaliński, Michał Glądalski, Marcin Markowski, Joanna Skwarska, Jarosław Wawrzyniak, Jerzy Bańbura, Piotr Zieliński

**Affiliations:** 1 Department of Experimental Zoology and Evolutionary Biology, Faculty of Biology and Environmental Protection, University of Łódź, Banacha 12/16, 90-237 Łódź, Poland; 2 Department of Ecology and Vertebrate Zoology, Faculty of Biology and Environmental Protection, University of Łódź, Banacha 12/16, 90-237 Łódź, Poland

**Keywords:** Wintering, Glucose, Great tit, Physiological condition, Ecology, Physiology, Zoology

## Abstract

Blood glucose concentration is one of the fundamental biochemical indices in the physiology of birds and other vertebrates. Since glucose serves as the main fuel for metabolic energy, its proper level in the blood serum is crucial to the individual in terms of physiological health, which leads to applying it as an ecophysiological indicator. Such an indicator may be especially useful during energetically demanding periods, such as winter at northern latitudes. Therefore, the main objective of this paper was to examine the variation in blood glucose in wintering the great tits (*Parus major)* in nine consecutive winters at two sites that differed with respect to the amount of human-provided food. We assumed that trophic conditions resulting from the use of artificial feeders that vary between sites across the years would affect the metabolic performance of birds and their physiological condition, which should be reflected in the blood glucose levels of wintering individuals. We showed that both females and males were characterised by significantly higher glucose levels at the study site, which was characterised by the high accessibility to human-provided food sources (forest clearing) than at the site with low and irregular artificial feeding. The difference remained distinct throughout most of the winter seasons of the study. We also revealed a substantial variation between seasons in blood glucose concentration in the case of male great tits. In general, we show that avian blood glucose can be used as an ecophysiological indicator of the trophic conditions of the environment in which individuals from small passerine species winter.

**Supplementary Information:**

The online version contains supplementary material available at 10.1038/s41598-025-86190-w.

## Introduction

Small passerines face considerable energetic challenges during winter. Low temperatures, particularly at night, food shortages during shortened winter days require elevated energy expenditures to keep birds alive and in good physiological condition. In classic papers on Parids Gibb^1,2^ presented evidence that there is a great shortage of food during winter as well as assessed that tits spend feeding 80–90% of the day and that a tit must find one average-sized insect every 2.5 s just to survive. It is of key importance to maintain metabolism and, consequently, thermogenesis at a high and stable level^3–6^. For the above reasons, the quality of wintering grounds in terms of the availability of adequate food sources is crucial for small wintering birds, including tits (*Paridae)*. Sudden changes in landscape use caused primarily by anthropogenic activity make some areas unsuitable or at least of lower quality for individuals spending winter there. Wintering under adverse conditions can increase the risk of starvation^7–9^. Therefore, even when individuals survive winter under worse trophic conditions (defined as both food supplies availability and quality in particular area in restricted time i.e. winter period), their physiological health can be weakened compared to their conspecifics wintering under optimal conditions. As a consequence, their breeding performance during the coming breeding season can be reduced. However, from several decades bird winter feeding has become very popular and in some countries such as Great Britain 75% households provide food for birds at some time of the year^10,11^. That relatively novel human activity profoundly changes conditions of wintering for several species, including tits, by creating locations with superabundant food sources in particular places (e.g. residential areas). At the same time, other areas without artificial feeders offer much worse conditions for wintering birds in terms of food availability.

Glucose is the basic energy-carrying substrate in vertebrates, including birds. The main role of glucose in the blood of birds is to provide energy to tissues during intense activities^12–14^. However, fatty acids and even proteins have been shown to be metabolised rather than glucose as a source of energy, especially during long-distance flight^15^. Birds maintain twice as high blood glucose levels as mammals of comparable body size^12^, and high glucose levels in birds reflect their high metabolic rates, at least in adult birds. Birds differ considerably with respect to blood glucose levels, which are usually higher in smaller species, reaching their maximum in hummingbirds that feed on nectar^16^. In general, excluding hummingbirds, birds can be roughly divided into two groups: passerines and non-passerine species, with metabolic rates over 60% lower in the latter group^17^. In addition to these general patterns within birds, there are several intrinsic and environmental factors that influence blood glucose concentration. Among intrinsic factors, body mass, sex, age, or breeding status are very important in this context^18–21^. However, environmental factors can affect blood glucose concentrations in a variety of ways. Plasma metabolite concentrations, including glucose, are known to be strictly related to environmental variables, since malnutrition in suboptimal habitats can be a strong environmental stressor that releases an endocrine response that affects glucose concentration in the blood^22,23^. However, the impact of particular environmental factors on the exact physiological mechanisms of glucose regulation in the blood of birds is still poorly understood^12^. Except for trophic conditions that are of crucial importance for metabolism, there are many different factors, such as weather conditions, predation rate or human activity in particular sites, that can influence glucose concentration in birds^24^.

Although data on glucose concentration are routinely collected in studies on flight metabolism and migration in many bird species^15,25,26^, relatively little is known about variation in blood glucose concentrations or other plasma metabolites in birds dwelling at different sites within restricted area such as the city (but see, e.g.^27^). In our previous study, we have shown that individuals of the great tit *Parus major* wintering in three sites that differ with respect to the availability of artificial food sources, an urban park, the city centre, and the deciduous forest, differ with respect to ketone body levels in the blood^28^. In addition to glucose, ketone bodies are one of the plasma metabolites related to energy metabolism, and their concentration in the circulating bloodstream indicates the nutritional state of an individual^29,30^. However, it should be stressed here that the study on ketones covered only one winter.

Taking into account that weather conditions vary from year to year, we conducted the study covering nine consecutive winters at two sites that differ completely with respect to the feeding activity by humans. Therefore, within the city of Łódź borders we chose Botanical Garden which is location poor in human supplied food with irregular feeding activity and a clearing in Łagiewniki Forest which constitutes rich in food, regular feeding site. Consequently, the main objectives of our study were (1) to examine whether there was a difference in the glucose concentration of the great tits between the two sites that differed with respect to the abundance of artificial food and (2) to show if there is a present year-to-year variation in blood glucose concentration during nine consecutive winters. We expected that in the artificial food rich site, blood glucose levels would be higher than in the artificial food poor site. We also expected that the glucose concentration would vary over the years as a result of variation in environmental conditions. In addition, as background for the main study objectives we showed body weight distribution in the two study sites across nine consecutive winters, for females and males separately, as well as the basic weather characteristics of all nine winters studied.

## Results

**Fig. 1:**
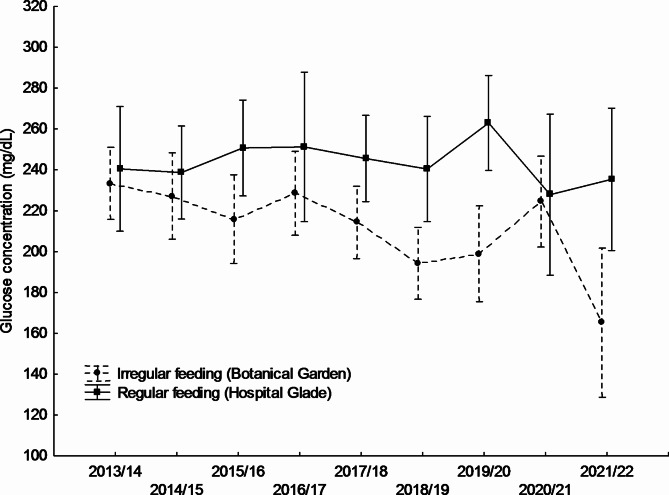
Annual variation in mean glucose concentration of great tit females in two study sites. Means ± 0.95 confidence intervals are shown.

**Fig. 2:**
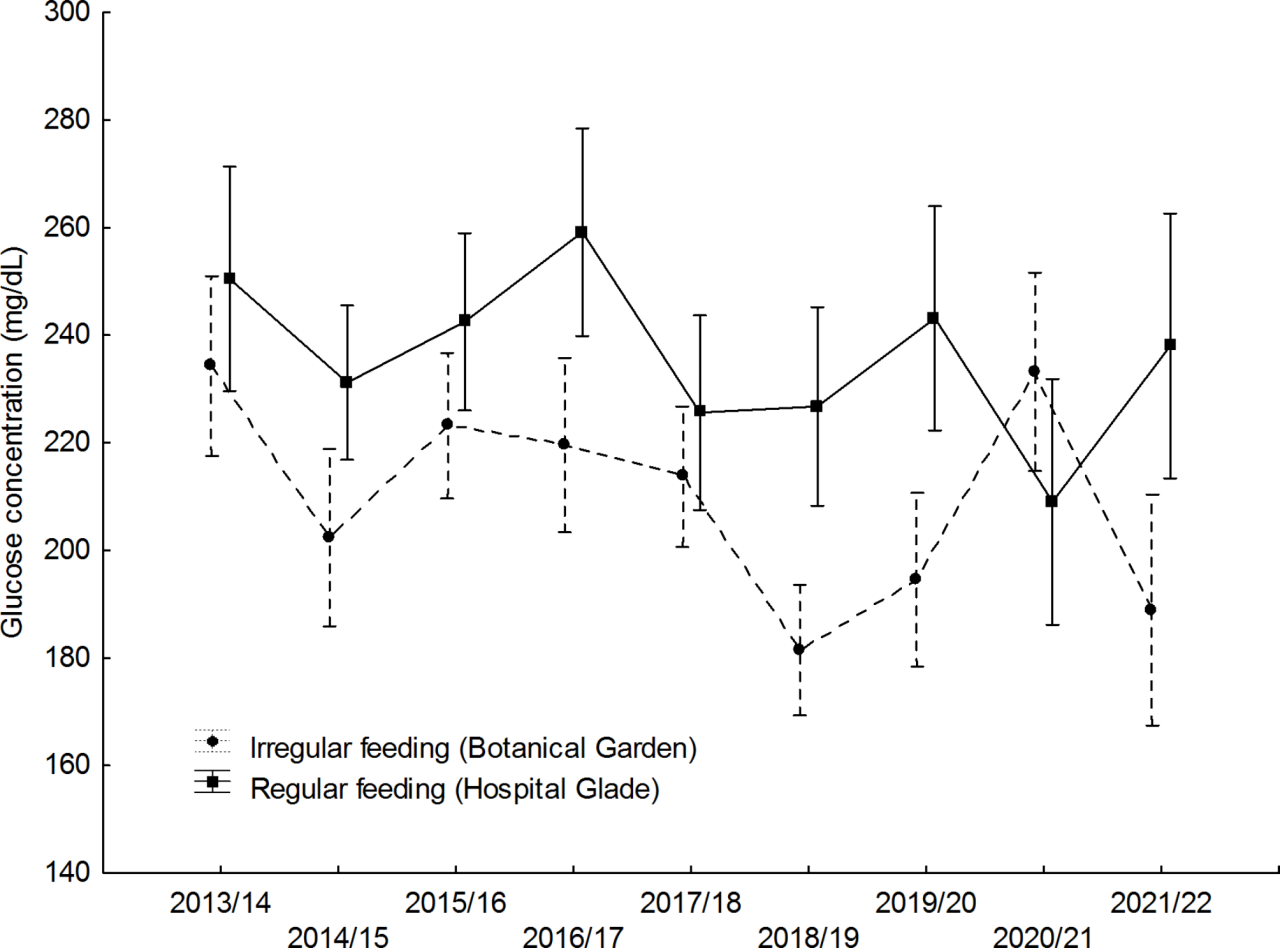
Annual variation in mean glucose concentration of great tit males in two study sites. Means ± 0.95 confidence intervals are shown.

**Fig. 3:**
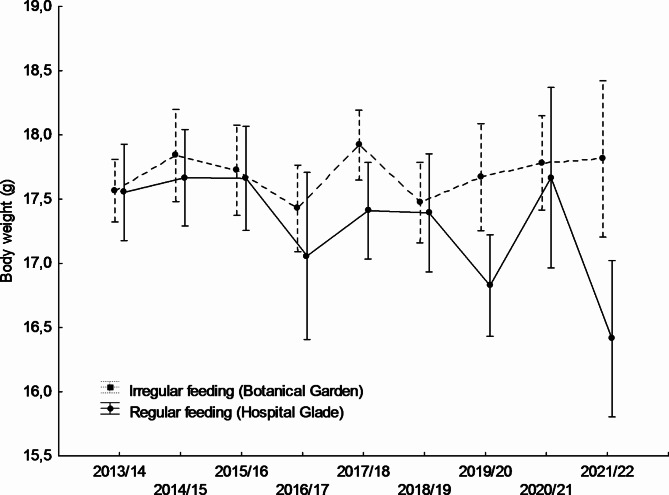
Annual variation in mean body weight of great tit males in two study sites. Means ± 0.95 confidence intervals are shown.

**Fig. 4:**
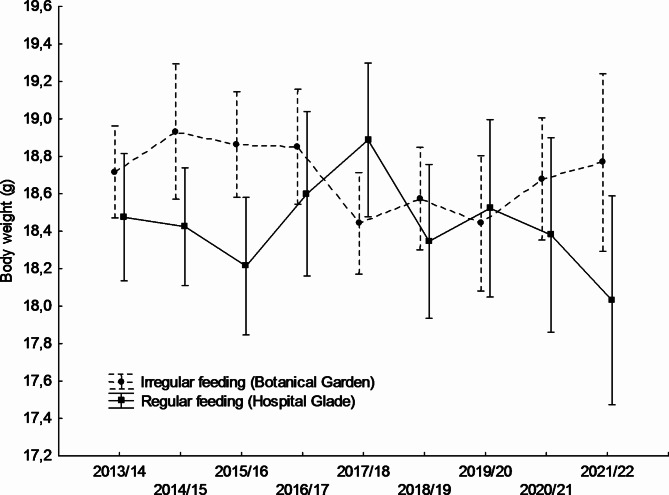
Annual variation in mean body weight of great tit males in two study sites. Means ± 0.95 confidence intervals are shown.

Mean blood glucose levels differed significantly between study sites and were higher in the Hospital Glade study area for both studied sexes (Table 1; Figs. 1 and 2). For females, the largest differences between study sites were recorded in winters 2015/16, 2018/19, and 2019/20 (Fig. 1), for males in turn in winters 2016/17, 2018/19, and 2019/20 (Fig. 2). Only in one winter 2020/21, the pattern reversed, with mean glucose concentration being higher in the urban park, but in the case of males only (Fig. 2), however, also in females mean values were almost equal that winter (Fig. 1). However, there was also a significant variation between years only in males (Table 1; Fig. 2). There was also a significant interaction between the study area and the year, but only in males (Table 1), in females this interaction was non-significant (F_8,304_ = 1.336; *p* = 0.225). The interactions between main effects and body weight covariate were non-significant and thus removed from the final models (GLM for males: feeding activity*body weight F_1,479_ = 0.523, *p* = 0.470; year*body weight F_8,479_ = 1,151, *p* = 0.328; GLM for females: feeding activity*body weight F_1,304_=0.873, *p* = 0.351; year*body weight F_8,304_ = 1,106, *p* = 0.359).Body weight covariate did not affect glucose levels in females or males. Mean body weight differed significantly between study sites and was higher in the Botanical Garden study area for both sexes studied (Table 2; Figs. 3 and 4). For females, the largest differences between the study sites were recorded in winters 2017/18, 2019/20, and 2012/22 (Fig. 3), for males, in turn, in winters 2014/15, 2015/16, and 2021/22. In two winters (2017/18, and 2019/20), the pattern was reversed, with mean body weight being higher in the Hospital Glade, but in the case of males only (Fig. 4). Furthermore, only for females body weight differed significantly between years with the mean highest value in winter 2014/15 and the lowest in winter 2021/22. Interactions between main effects were non-significant (GLM for females: F_(8,357)_ = 1.7; *p* = 0.087 and for males: F_(8,591)_ = 1.8; *p* = 0.069).

**Table 1:** Summary of blood glucose concentrations in the two separate GLMs in females (top) and males (bottom).

Factor	Df	F	*P*
Females			
Intercept	1; 304	5.666	**0.018**
Feeding activity (study site)	1; 304	28.083	**< 0.001**
Year	8; 304	1.803	0.076
Body weight	1; 304	3.123	0.078
Males			
Intercept	1; 479	21.279	**< 0.001**
Feeding activity (study site)	1; 479	35.781	**< 0.001**
Year	8; 479	4.075	**< 0.001**
Body weight	1; 479	2.167	0.141
Feeding activity (study site) * Year	8; 479	2.827	**0.004**

The effects of the feeding activity (study site), year, and individual body weight as a covariate are given. Only for the males the interaction between main factors is significant and thus presented in the table. Only final models are presented.

Significant values are in [bold].

**Table 2:** Summary of body weight in the two separate GLMs in females (top) and males (bottom).

Factor	Df	F	*P*
Females			
Intercept	1; 357	109030.6	**< 0.001**
Feeding activity (study site)	1; 357	14.0	**< 0.001**
Year	8; 357	2.1	**0.035**
Males			
Intercept	1; 591	162082.2	**< 0.001**
Feeding activity (study site)	1; 591	8.3	**0.004**
Year	8; 591	0.6	0.780

The effects of the feeding activity (study area) and year are given.

Significant values are in [bold].

## Discussion

We measured glucose levels in a small passerine species, the great tit, in nine consecutive winter seasons. As expected, mean glucose levels were significantly higher in both females and males in the study site that was rich in artificial food in virtually all seasons studied. Furthermore, the glucose concentration varied significantly between years, but in males only; however, in particular seasons, it tended to be parallel between the two sites for both sexes. Our results suggest that the trophic conditions in the wintering grounds were relatively poorer in the urban park study area.

Glucose, as the main energy source for working muscles in birds, must be kept at a possibly stable level in the blood stream. Under typical conditions, glucose concentration reflects the actual level of carbohydrate ingestion and increases or decreases with the satiation state of an individual^12,13^, and therefore blood glucose concentration is strictly related to the food supply available to individuals. For this reason, glucose concentration can serve as an index of the physiological nutritional state of an adult individual and can indirectly indicate the trophic quality of a particular environment. The results reported in the present study show such an exact pattern. Birds that winter in the Hospital Glade are characterised by higher mean glucose concentrations.

Small birds that winter in northern latitudes must cope with severe environmental conditions. They need to store enough energy to survive long night periods when temperatures frequently fall well below zero and the foraging time is restricted to relatively short daytime periods^31–34^. The great tit is a small species that is widely distributed throughout the western Palearctic^35^. In Central Europe, they are mainly sedentary; however, dispersing flocks of mainly juveniles wander in a random manner, usually within rather short distances. Furthermore, individuals from the northern part of the species range migrate further south during autumn-winter, especially when weather conditions are particularly severe^36,37^. Therefore, in winter, great tits are found in a variety of habitats, from riverine forests and reedbeds, forest edges, suburban areas, and city centres^38^ (own obs.). All these types of environment differ with respect to various characteristics, but without a doubt one of the most important features is food quality and availability. In rural or in general less anthropogenic altered areas, great tits during winter feed mainly on insect eggs or pupae, seeds of beech or hazel^35^, but in urban areas they readily use relatively novel and often superabundant food sources in the form of feeders supplied with different kinds of seeds or fat of animal origin^11^ (own obs.). We assume that the pattern we found results mainly from differences in this abundance of anthropogenic food between the two sites. At the Hospital Glade, 6–8 feeders operate during the autumn-winter period throughout all years analysed in this study. Unlike Hospital Glade, feeders in the Botanical Garden are almost absent, with the exception of a small number of feeders located sparsely on the eastern edge of the Botanical Garden in the residential house area. In our previous study on wintering great tits, we showed that blood ketone levels were significantly higher in the Botanical Garden than in the clearing in Łagiewniki and city centre^28^. Since high ketosis is indicative of fasting and results from fatty acid and protein metabolism rather than glucose^13^, it suggests that birds are more vulnerable to food shortages in the parkland area. The study on ketones was based on an analysis on one winter season alone, but the results were clear for both females and males.

In the current study, in eight out of nine winter seasons, glucose levels were higher in the Hospital Glade for both females and males. It is not clear why during one winter season (2020/2021) this pattern was reversed. It is possible that for unknown reasons the regularity of supplying the feeders with food in the Hospital Glade was interrupted. Taking into account the occurrence of the COVID-19 pandemic during 2020–2021, it could be a possible cause of interruption in the continuity of food delivery in the case of the hospital direct surroundings in the Hospital Glade, where most local feeders operate. Nevertheless, our present study shows that the Botanical Garden in most years offers worse trophic conditions for wintering great tits. We suppose that the Hospital Glade, which is a kind of artificial clearing with feeders operating throughout winter and surrounded by a coherent forest habitat, may be attractive to individuals that winter in this area. What is important, at the Hospital Glade each winter, we observe mixed flocks of tits (in addition to great tits, and blue tits also marsh tit *Poecile palustris*) mainly within the close proximity to the feeders or at the forest edges and birds are rarely seen in the depth of the vast forest area (own obs.). It supports the suggestion that the extensive forest itself is not very attractive for wintering tits and probably the high density of feeders is what attracts the birds most. By contrast, the Botanical Garden is a relatively large green area within the city of Łódź, poor in feeders. Paradoxically, the density of wintering parids (mainly great tits and blue tits) is higher in the urban park than in the Hospital Glade (own obs.), but it is in line with our results, since more intensive competition for food may strengthen the effect of food shortages in the Botanical Garden but not in the Hospital Glade. Our long-term parid ringing study at both study sites during the winter seasons and ringing almost all local birds during breeding season shows that most individuals are migrants (up to 95%) with a minority of local birds. Simultaneously our observations during several consecutive winters at the feeding stations and their vicinity shows that there are more migrants in the Botanical Garden than in the Hospital Glade in the forest with the proportion approximately 5 to 1 in favour of the parkland (own obs.). Long-term migrants rely on fat reserves as their main energy source and, after a long flight, they have to forage intensively to refuel the reserves^26,39^. It is possible that for migrating tits the vast green parkland area within the city borders can be attractive, but may simultaneously act as a kind of ecological trap since it offers a relatively small number of stable food sources.

We cannot exclude other factors, such as predation, that can negatively affect the satiation state of wintering individuals. Some clues on that issue can deliver weight distribution of individuals females and males great tit in both study sites. In most of the seasons, birds were heavier in the Botanical Garden. It may seem surprising that in the location with easy access to feeders, individuals are lighter than expected. A possible explanation may lie in the extent of predation risk between sites. There is a much higher predation pressure exerted by sparrowhawks *Accipiter nisus* in the Hospital Glade than in the Botanical Garden (own. obs.). Earlier studies show that birds gain less fat reserves than expected, which implies that there is a cost of being fat. This cost can result from the fact that increased fat load reduces manoeuvrability, thus increasing the risk of predation^40–42^. This interpretation is in tune with the main result of this study, since it is known that high glucose levels in the blood can be to some extent stress-induced. We have found such a pattern during the breeding season in nestlings for both great tits and blue tits *Cyanistes caeruleus*^18^,^24^. In our study system, where the forest area during the breeding season was richer in terms of caterpillar abundance, mean glucose levels were lower there than in the Botanical Garden in both tit species. We showed that for small altricial bird nestlings, elevated glucose levels are a result of environmental stress caused by malnutrition. It clearly shows that blood glucose levels tell us slightly different stories in the case of rapidly growing nestlings and for adult individuals wintering in demanding environments in which they have to work hard during a short day to gather enough food items just to keep themselves alive.

We also found considerable year-to-year variation tending to be parallel between the study sites within particular seasons. It is difficult to interpret this result, since a great variety of different factors may impact the physiology of birds. Weather characteristics may play some role here; however, it seems that this study it is not the case since all winter seasons were characterised by relatively mild thermal conditions and small snow cover. However, extremal weather conditions may influence to some extent mean glucose levels, since during winter 2016/17 when the precipitation sum was at its highest level mean glucose was highest in the forest, but in the case of males only. It suggests that heavy rain is likely to influence energy metabolism in wintering tits, probably through shifting the feeding behaviour of birds. Therefore, it seems that the effect of the weather variables per se on blood glucose concentrations is rather subtle and does not influence energy metabolism directly. We cannot also exclude the possibility that the regularity of feeding at Hospital Glade which is out of our control could be interrupted to some extent.

## Conclusion

In conclusion, we emphasise the role of glucose concentration as a reliable index of condition in wintering great tits. We show that spatial variation in blood glucose constitutes a distinct pattern with glucose levels significantly higher at the Hospital Glade than at the urban park study site. We argue that this pattern is mainly caused by trophic conditions during the winter seasons. The high availability of well supplied feeders in Hospital Glade creates suitable conditions for wintering passerines, however the higher predation risk can generate some energetical costs together with stress-response Our results suggest that for the great tit, the choice of a wintering place is of crucial importance, since the trophic conditions there profoundly influence the physiological health of individuals. This, in turn, has consequences for the fitness of a bird and its chances of survival for the subsequent breeding season. We also show substantial year-to-year variation which probably results from interruptions in feeders refuelling with food rather than the variability in weather conditions. In summary, our study shows that the blood glucose level, which is relatively easy to obtain directly in the field, can be used as an indirect indicator of the trophic quality of the environment for wintering small passerine species and, therefore, is suitable in a variety of ecological research. However, it should be stressed that the overall effect of supplementary feeding on avian metabolome is complex and strongly context-dependent (see e.g.^43^). Therefore, future studies should seek connections between various avian metabolic characteristics in different ecological contexts.

## Methods

### Study area and field procedures

Data were collected in the nine consecutive winter seasons: 2013/2014–2021/2022. The study was carried out from the beginning of December to the end of February each winter. The study areas were winter bird feeding stations located around the city of Łódź in two different locations: an urban park and a clearing in deciduous forest. The poor feeding site (51°45′N; 19°24′E) is located in the SW part of Łódź in the Botanical Garden. The Botanical Garden is adjacent to the highly urbanised area of the city with numerous residential properties from the south, southeast, and north. The Garden is closed to visitors during late autumn and winter. The feeding station in the botanical garden was located close to its western border with an adjacent area of wastelands covered mainly by low shrubs and low herbaceous vegetation. This feeding station was provided with food (sunflower seeds) two days before the day of bird trapping, and no other feeding sites were located within a radius of approximately 0.7 km.

The rich feeding site is located in a forest clearing along the tarmac road that crosses the Łagiewniki Forest (51°50′N; 19°29′E). In the clearing there is a lung disease hospital, a wildlife rescue centre, a municipal police office, and an ecological education centre. Collectively, they constitute a compact glade within a large area of predominantly deciduous and mixed forest (of a total area of approximately 1250 ha) that is close to the NE suburbia of Łódź. Since the hospital area is the largest we will subsequently call this study site the ‘Hospital Glade’. At the Hospital Glade, 6–8 feeding stations are operated each winter, most of them in the hospital area. At Hospital Glade, our bird trapping site and feeding station is located next to the building of the ecological education centre. Also, this feeding station was provided with food in the same manner as in the Botanical Garden.

During the study, birds were caught in a standardised manner in ornithological mist-nets. Two 5-m-long mist nets were used during each trapping in both study areas. Two-three days before each trapping, a firm amount of sunflower seeds was placed at each site in standard bird feeders made of plastic containers. Trapping was carried out 6–7 times in each study area and in each winter, from the beginning of December to the end of February. All individuals were captured within a relatively narrow time window during the day (9.00–13.00) to avoid possible fluctuations in glucose levels due to the diurnal schedules of the wintering individuals. During the study, 817 individuals were sampled: 197 females in the Botanical Garden and 119 in the Hospital Glade, and 300 and 201 males respectively. All individuals were ringed, weighed to the nearest 0.1 g using an electronic scale, and their sex was determined^44^. Subsequently, each individual was bled from the ulnar vein and a sample of approximately 5 µL blood was taken directly to HemoCue cuvettes and analysed in the field for glucose concentration using a portable HemoCue 201 + photometer (HemoCue AB, Angelholm, Sweden). The birds were released immediately after sampling.

### Weather conditions

The nine consecutive winter seasons of 2013/2014 to 2021/2022 were characterized by mild weather conditions with average temperatures in three winter months above 0 °C in 7 out of 9 winter seasons. The mean minimum daily temperatures were the lowest in the 2016/17 winter season and the highest in 2019/20 (Table 3). The sum of days with snowfall and the sum of days with a mean daily temperature below 0 °C were at similar levels in the nine winters (Table 3). Precipitation was at low or medium levels (with the exception of 2016/2017 and heavy rainfall) with rain dominating snowfall (Table 3). On bird trapping days, the average temperatures per particular winter ranged from − 1.7 °C (coldest winter) to 2.8 °C (warmest winter). We checked the correlations between mean glucose levels in a particular winter (separately for both sexes and both sites) and weather characteristics (mean minimum daily temperature, sum of days with snowfall, sum of days with mean temperature below 0°, precipitation sum). All the correlations were non-significant with p values ranging from 0.123 to 0.993, and r absolute values ranging from 0.0036 to 0.5521. More exact values of mean monthly temperatures, sum of rainfall, and snowmelt are given in Appendix A.

**Table 3:** Winter (December-February) weather characteristics (mean minimum daily temperature, sum of days with snowfall, sum of days with mean temperature below 0°, and precipitation sum) in nine consecutive study seasons.

Season	Mean minimum temperature (°C)	Days with snowfall	Days with mean temp. below 0 °C	Precipitation sum (mm)
2013/2014	-2.06	18	53	20.6
2014/2015	-1.87	35	52	91.6
2015/2015	-1.19	25	44	109.0
2016/2017	-4.87	36	71	453.4
2017/2018	-2.88	37	64	106.7
2018/2019	-2.52	39	61	131.8
2019/2020	-0.28	19	44	93.2
2020/2021	-3.46	35	66	77.2
2021/2022	-2.15	29	56	165.9

### Data analysis

Variation in glucose concentration was analysed using general linear models (GLM). It was tested whether the category of feeding activity (permanent feeding vs. irregular feeding) and season affected glucose concentration. Feeding activity category (study site) and season were included as fixed factors in these models, both as main effects and as interactions with each other. Since we expected that body weight may covary with glucose concentrations, we added this trait as a covariate. The analyses were performed with glucose concentration as a dependent variable. Since males and females differ with respect to their body masses and physiological responses to external factors, we analysed the sexes in two separate models.

The initial models for each sex included the dependent variable (glucose concentration) in relation to main effects: the covariate (body mass), the fixed factors (feeding activity category and year); three two-way interactions between the main effects were also included. The initial models were simplified by one-by-one backward removing non-significant interactions, beginning from the least significant one to get the final model, with all main effects being retained^45^.

The body weight distribution was analysed using a separate one-way GLM for each sex with feeding activity (study site) and year included as fixed factors. Both these models included the main effects and their interaction with each other. The interactions proved to be non-significant and were removed. The GLM modelling was performed using Statistica software^46^.

## Electronic supplementary material

Below is the link to the electronic supplementary material.

Supplementary Material 1

## Data Availability

The data set used for calculations can be found online at https://figshare.com/articles/dataset/Blood_glucose_of_wintering_great_tits/26334721?file=47790727.
