# Supplementary material for: Long term study on blood glucose levels in wintering great tits Parus major in sites differing in artificial food availability
Source: Sci Rep. 2025 Jan 20;15:2519. doi: 10.1038/s41598-025-86190-w (PMC11747514; doi:10.1038/s41598-025-86190-w)
Supplement: Supplementary file 1 — Supplementary Material 1 [file 41598_2025_86190_MOESM1_ESM.docx]

**Appendix**

**Supplementary table 1**. Mean temperatures (°C) in the winter months (December-February) during the study period.

| Month  Winter | December | January | February | Year mean December - February |
| --- | --- | --- | --- | --- |
| 2013/2014 | 2.3 | -1.7 | 2.7 | 1.1 |
| 2014/2015 | 1.2 | 1.4 | 0.8 | 1.1 |
| 2015/2016 | 5.4 | -2.5 | 3.6 | 2.2 |
| 2016/2017 | 1.1 | -4.5 | -0.9 | -1,4 |
| 2017/2018 | 2.2 | 1.0 | -3.3 | 0,0 |
| 2018/2019 | 1.6 | -1.7 | 2.6 | 0.8 |
| 2019/2020 | 3.1 | 1.9 | 4.0 | 3.0 |
| 2020/2021 | 1.9 | -1.5 | -1.0 | -0.2 |
| 2021/2022 | -0.9 | 0.8 | 3.2 | 1.0 |

**Supplementary table 2**. The sum of rain and snowmelt (mm) in the winter months (December-February) during the study period.

| Month  Winter | December | January | February | Year sum December - February |
| --- | --- | --- | --- | --- |
| 2013/2014 | 20.6 | 10.5 | 12.5 | 43.6 |
| 2014/2015 | 56.6 | 38.1 | 8.4 | 103.1 |
| 2015/2016 | 26.6 | 26.9 | 57.4 | 110.9 |
| 2016/2017 | 83.6 | 250.2 | 120.2 | 454.0 |
| 2017/2018 | 42.7 | 58.2 | 5,8 | 106.8 |
| 2018/2019 | 63.0 | 42.9 | 35.1 | 141.0 |
| 2019/2020 | 26.4 | 29.5 | 52.3 | 108.2 |
| 2020/2021 | 87.1 | 35.3 | 21.3 | 143.7 |
| 2021/2022 | 28.0 | 85.3 | 52.6 | 165.9 |

**Supplementary table** **3**. Mean temperatures (°C) on bird trapping days only during the study period.

| Winter | Temperature |
| --- | --- |
| 2013/2014 | 2.1 |
| 2014/2015 | 1.0 |
| 2015/2016 | 1.2 |
| 2016/2017 | -1.7 |
| 2017/2018 | 2.8 |
| 2018/2019 | 0.1 |
| 2019/2020 | 2.3 |
| 2020/2021 | 1.5 |
| 2021/2022 | -1.3 |
